# The Nucleoskeleton: Crossroad of Mechanotransduction in Skeletal Muscle

**DOI:** 10.3389/fphys.2021.724010

**Published:** 2021-10-15

**Authors:** Shama R. Iyer, Eric S. Folker, Richard M. Lovering

**Affiliations:** ^1^Department of Orthopaedics, University of Maryland School of Medicine, Baltimore, MD, United States; ^2^Department of Biology, Boston College, Chestnut Hill, MA, United States; ^3^Department of Physiology, University of Maryland School of Medicine, Baltimore, MD, United States

**Keywords:** nucleus, intermediate filaments, lamins, mechanotransduction, aging, muscle disease

## Abstract

Intermediate filaments (IFs) are a primary structural component of the cytoskeleton extending throughout the muscle cell (myofiber). Mechanotransduction, the process by which mechanical force is translated into a biochemical signal to activate downstream cellular responses, is crucial to myofiber function. Mechanical forces also act on the nuclear cytoskeleton, which is integrated with the myofiber cytoskeleton by the linker of the nucleoskeleton and cytoskeleton (LINC) complexes. Thus, the nucleus serves as the endpoint for the transmission of force through the cell. The nuclear lamina, a dense meshwork of lamin IFs between the nuclear envelope and underlying chromatin, plays a crucial role in responding to mechanical input; myofibers constantly respond to mechanical perturbation via signaling pathways by activation of specific genes. The nucleus is the largest organelle in cells and a master regulator of cell homeostasis, thus an understanding of how it responds to its mechanical environment is of great interest. The importance of the cell nucleus is magnified in skeletal muscle cells due to their syncytial nature and the extreme mechanical environment that muscle contraction creates. In this review, we summarize the bidirectional link between the organization of the nucleoskeleton and the contractile features of skeletal muscle as they relate to muscle function.

## Introduction

Intermediate filaments (IFs) contribute to force transmission, cellular integrity, and signaling in skeletal muscle. IFs are flexible, rod-shaped fibers averaging 10 nm in diameter, a size that is “intermediate” between microfilaments (7–8 nm) and microtubules (25 nm). IFs are classified into major families, with types I–IV localized in the cytoplasm, type V comprised of the lamins inside the nucleus, and type VI comprised of neural and lens proteins. In mature skeletal muscle, the IF cytoskeleton is composed predominantly of type III IF proteins, with the muscle-specific protein desmin being the most abundant. Desmin is an intracellular protein linking individual myofibrils laterally to each other and is important for the transmission of active and passive forces within the cytoskeleton (Shah et al., 2004). Other IFs in skeletal muscle include type IV IF proteins, such as synemin, paranemin, syncoilin and nestin, and IF type I and II keratins, all of which are important in cell integrity and cytoskeletal transmission of force (McCullagh et al., 2007; Lovering et al., 2011; Garcia-Pelagio et al., 2015).

In addition to the cytoplasmic IF network, the nuclei contain a network of lamin IF proteins. Like their cytoplasmic counterparts, the nuclear lamins are an important determinant of nuclear stiffness and are crucial in maintaining the integrity of the nuclei in the mechanically intense environment of a contracting muscle (Swift et al., 2013; Stephens et al., 2017). Many studies have investigated how forces initiated at the sarcolemma are transmitted throughout the cytoskeleton to activate downstream cellular responses (i.e., mechanotransduction). The signaling cascades that respond to changes in extracellular and intracellular mechanics have garnered the most focus (Tidball, 1991). However, recent studies show that forces acting on the cell, and changes in stiffness within the cell, can be transmitted directly to the nucleus and change the structure of the nuclear lamina and the associated chromatin (Stephens et al., 2017, 2019). Thus, the nucleus can act as an internal mechano-sensor in muscle cells (myofibers) (Navarro et al., 2016).

Although most eukaryotic cells have a single nucleus, myofibers are multinucleated. To support the large cytoplasm in the skeletal myofiber, the nuclei are evenly positioned along the cell periphery to maximize the distance between nuclei (Bruusgaard et al., 2006). This even spatial distribution of nuclei minimizes transport distances, such that each nucleus regulates the gene products in a fixed volume of a muscle fiber, a concept known as the myonuclear domain (Hall and Ralston, 1989). There is some flexibility in the size of myonuclear domains, but whether nuclear accretion is necessary for, versus the result of, increased muscle fiber size is less clear (Murach et al., 2018). Each nucleus contributes to the organization of the myofiber cytoskeleton, but importantly the nuclei throughout a single fiber also act as cellular mechano-sensors (Cho et al., 2017). Nuclear spatial distribution distributes the “centers” that organize the cytoskeleton and mechanical hubs that are critical mechanical sensors and responders. These hubs are then integrated with the force-producing myofibrils through the cytoplasmic IF network (Roman and Gomes, 2018). Thus, understanding the individual contributions of distinct IF networks and how these distinct pathways interact is critical to understanding muscle weakness with aging and disease.

## Lamina and Nuclear Envelope

**FIGURE 1:**
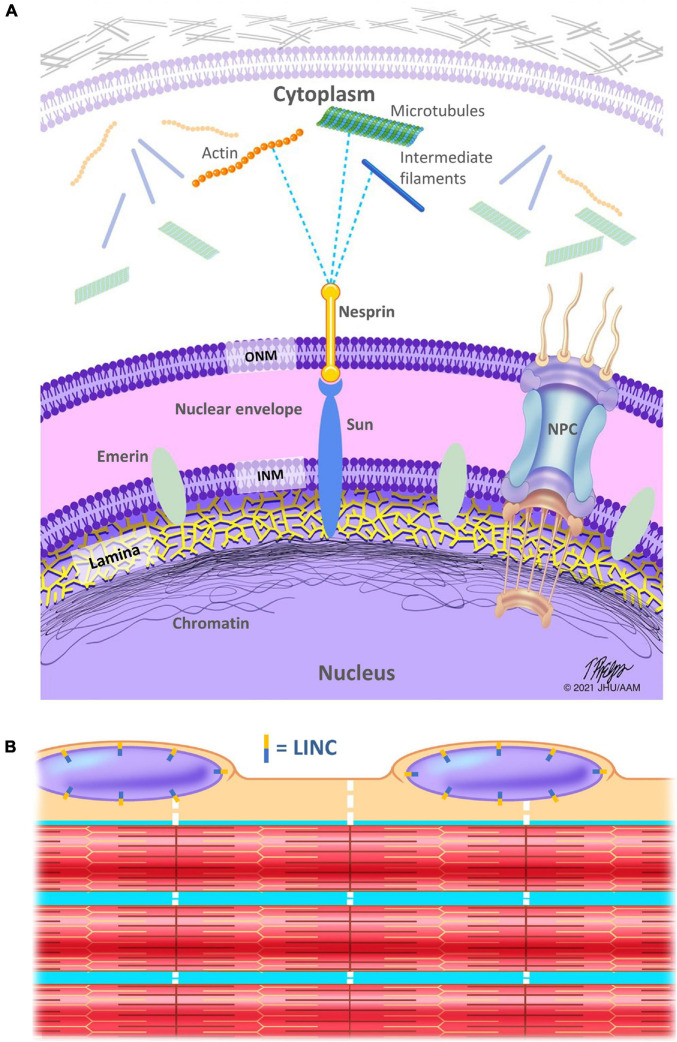
Schematic representation of the linker of nucleoskeleton and cytoskeleton (LINC) complex. **(A)** The nuclear lamina is a meshwork of intermediate filaments (lamins), localized on the inner aspect of the inner nuclear membrane and helps to stabilize the nuclear envelope. The lamina is in direct contact with the chromatin, which is denser at the nuclear periphery. SUN proteins connect the inside of the nucleus to nesprins, which serve as a link between the nucleus and the myofiber cytoskeleton (including but not limited to microtubules, the actin, and desmin IFs), directly or indirectly via plectin, a cytoskeletal link in the cytoplasm. INM, inner nuclear membrane; ONM, outer nuclear membrane; NPC, nuclear pore complex. **(B)** Schematic showing only a few of the hundreds of myofibrils within a single muscle fiber (aka myofiber). Nuclei are located along the periphery of myofibers. Note that the LINC complex is located circumferentially around the nuclear membrane (aka nuclear envelope). Dotted white lines indicate the cytoskeletal IFs that connect the myofibrils to each other, to the sarcolemma, and to the nuclei.

The nucleus serves as the instruction manual for cells, containing the vast majority of the genome and the site of gene regulation. Thus, how it interprets stress to alter gene expression and its position to deliver cellular materials clearly contributes to cell behavior. Like most cell organelles, the nucleus is surrounded by a membrane. However, the nuclear envelope (NE) is composed of a double bilayer lipid membrane that forms a barrier between the nuclear interior and the cytoplasm (Figure 1). This NE consists of an outer nuclear membrane (ONM) and the inner nuclear membrane (INM) that are separated by the small (∼40 nm wide) perinuclear space. The two membranes are fused at the nuclear pore complexes (NPCs), which form channels responsible for nucleocytoplasmic trafficking.

The linker of nucleoskeleton and cytoskeleton (LINC) complex is a series of proteins that physically links the nucleoskeleton to the cytoskeleton and allows for transmission of force from the cytoplasm to the nucleus for many functions including nuclear spacing and gene regulation (Luxton and Starr, 2014). In mammals, nesprins (nuclear envelope spectrin repeat proteins), which contain a KASH domain, span the ONM and interact with microtubules, actin, and IFs on the cytoplasmic side of the nuclear envelope. SUN proteins (Sad1 and UNC-84 domain containing proteins) span the INM and interact directly with both the nuclear lamina and chromatin on the nucleoplasmic side of the nuclear envelope. Nesprins, specifically their KASH domains, interact with the SUN domain proteins within the perinuclear space (space between the INM and ONM). Thus, these proteins directly link protein networks that are critical to mechanotransduction. Furthermore, these proteins interact and are responsive to the mechanical environment (Jahed et al., 2021), adding a layer of complexity and tuning to this integrated network. This complexity is particularly important in muscle where LINC complex interactions are critical to maintain spacing between the two nuclear membranes (Cain et al., 2014).

Within the nucleus, the lamina is composed of ∼3.5 nm tetrameric lamin filaments and is approximately ∼14 nm thick. The lamina lines the INM, connects chromatin to the nuclear periphery, and directly interacts with transcription factors to regulate gene expression (Turgay et al., 2017). In addition, the NPCs, which allow for selective transport between the nucleus and the cytoplasm, interact with the LINC complex, the lamina, the cytoskeleton, and the chromatin to regulate gene expression (D’Angelo, 2018). Thus, the complex of proteins that regulate the movement of proteins in and out of the nucleus contacts the regulators of gene expression and the primary mechanotransduction networks. Furthermore, the NPC itself is mechanically responsive demonstrating the ability to expand in response to physical cues (Fichtman and Harel, 2014).

The lamin family consists of the A-type lamins and the B-type lamins, and are expressed as products of three differentially regulated genes. Lamin A, C, and less abundant, AΔ10 and C2, are all splice-isoforms of the *LMNA* gene. Lamin B1 is encoded by *LMNB1* and Lamin B2 and B3 are encoded by *LMNB2* gene. Different lamin isoforms interact to form the lamina and have many binding partners, including chromatin, LINC complex proteins and NPCs. Similar to other IF family members, lamins are composed of a α-helical rod domain with a short N-terminal head and a long tail domain containing an Ig-fold. The rod and head domains are essential for lamin assembly, but other regions are also important for proper protein function. The tail domain with the globular Ig-fold contains most of the interaction sites for lamin-binding partners. Lamins govern numerous biological functions, both biophysical and biochemical (Turgay et al., 2017). This includes determination of nuclear size, shape, stiffness (Dahl et al., 2004), regulation of transcription factors and chromatin, and control of cell polarization and migration (Davidson and Lammerding, 2014). However, the relative importance of individual lamin networks remains an open question highlighted by the segregation of lamin A and lamin B proteins within an integrated meshwork (Guilluy et al., 2014).

Inside the nucleus are threads of chromatin composed of DNA and associated proteins characterized as either open, transcriptionally active euchromatin or condensed, inactive heterochromatin. Chromatin associated with the NE has been described as silent chromatin, which interacts with the nuclear lamins, while active chromatin interacts with the nuclear pore complex proteins (Kalverda et al., 2008). INM proteins interact with the lamina and/or chromatin in a tissue-specific manner. Although the interactions between lamins and chromatin are critical to the organization of each, the network of chromatin within the nucleus provides a functionally separate mechanotransduction mechanism (Turgay et al., 2017; Dos Santos and Toseland, 2021).

## Nuclear Envelope and Nuclear Mechanotransduction

Mechanical forces are conducted through myofibers and into nuclei to regulate muscle development, hypertrophy, and homeostasis. One mechanism that allows myofibers to respond to the constant mechanical force they are subjected to is the regulation of protein levels. The stability, assembly and transcription of the nuclear lamins, LINC complex, and the NPCs are required for proper mechano-signaling, as all of these components respond to mechanical stress (Dahl et al., 2008). Although much remains to be learned, there is reason to expect that the changes in protein levels are a direct response to cellular mechanics. Extracellular and cytoplasmic forces are transmitted across the nuclear envelope to the nuclear interior, where they can cause deformation of chromatin and nuclear bodies (Guilluy et al., 2014). Furthermore, the proportion of phosphorylated lamin A, a proxy for the percentage of lamin A that is incorporated into the network, is dependent on cellular mechanics (Swift and Discher, 2014). Because gene expression is dependent on chromatin organization (Nava et al., 2020), which can be affected by interaction with the lamina or directly by the application of force, it is likely that muscle development and homeostasis are closely tied to the mechanical state of the cell (Bronshtein et al., 2015). The nuclear lamina is crucial to transducing mechanical signals to the nucleus and plays an important role in DNA organization, DNA repair, and transcriptional regulation [reviewed in Gruenbaum and Foisner (2015)].

Beyond its gene regulation, the nuclear lamina provides critical structural support that protects the nucleus against nuclear rupture and damage (Cho et al., 2019). Additionally, the nuclear lamina serves to receive mechanical signals and thus has been called a molecular “shock absorber” of the nucleus (Osmanagic-Myers et al., 2015). Mechanical stiffness and viscosity of nuclei are dependent on A-type lamins, while deformability and elasticity of nuclei are dependent on B-type lamins. Protein expression of lamin A scales with increasing tissue stiffness (Swift et al., 2013). In addition, increased mechanical load of the nucleus suppresses lamin A/C phosphorylation, with degradation of lamin A/C, downstream to phosphorylation (Buxboim et al., 2014).

## Nuclear Envelope in Disease and Aging

Changes to the nuclear envelope and its associated proteins can result in unstable regulation of the mechanotransduction pathway in response to mechanical stress, which drives phenotypic changes in muscle, such as weakness seen with sarcopenia and muscular dystrophies (Lacavalla et al., 2021). Mutations in the *LMNA* gene are associated with diseases having a wide range of phenotypes, including striated muscle dysfunction, and are collectively referred to as “laminopathies,” this includes effects on aging, for example Hutchinson-Gilford progeria (Table 1). Mutations in the genes encoding LINC complex proteins are also associated with a wide range of skeletal muscle dysfunctions, many of which have phenotypes that overlap with those of laminopathies (Bonne and Quijano-Roy, 2013). Interestingly, we recently reported reduced expression of lamin-B1 in aged skeletal muscle (Iyer et al., 2021). Synaptic nuclei (nuclei anchored underneath the neuromuscular junction) in aged skeletal muscle have reduced expression of *LMNA* gene (Gao et al., 2020). We have also observed changes in gene expression of LINC complex proteins in murine models of Duchenne muscular dystrophy (Iyer et al., 2016). However, the mechanisms in this reduction and its effect on aging and diseased skeletal muscle remain to be elucidated.

**TABLE 1:** Diseases associated with mutations in genes for lamin proteins and proteins associated with the LINC complex.

**Disease**	**Gene**	**Protein**	**Main clinical features**
**Emery-Dreifuss muscular dystrophy 2, autosomal dominant**	LMNA	Lamin A and C	Formerly known as Limb-girdle muscular dystrophy type 1B; muscle weakness and atrophy; neck, elbow and Achilles tendon contractures; cardiac conduction defects and dilated cardiomyopathy
**Emery-Dreifuss muscular dystrophy 3, autosomal recessive**			Muscle weakness and atrophy; neck, elbow and Achilles tendon contractures; cardiac arrhythmias
**LMNA-related congenital muscular dystrophy**			Severe muscle weakness and atrophy; joint contractures
**Dilated Cardiomyopathy, 1A**			Dilated cardiomyopathy
**Charcot-Marie-Tooth disease, type 2B1**			Muscle weakness and atrophy; peripheral neuropathy; reduced tendon reflexes
**Heart-hand syndrome**			Limb deformities; dilated cardiomyopathy; tachyarrhythmia; progressive sinoatrial and atrioventricular conductive disease
**Hutchinson-Gilford progeria**			Premature aging
**Lipodystrophy, familial partial, type 2**			Abnormal subcutaneous adipose tissue distribution; metabolic abnormalities
**Malouf Syndrome**			Hypogonadism; dilated cardiomyopathy
**Mandibuloacral dysplasia**			Craniofacial and skeletal abnormalities; growth retardation; abnormal adipose tissue distribution
**Restrictive dermopathy, lethal**			Early neonatal death; tight, rigid skin with erosion and fissures; joint contractures; superficial vessels; facial dysmorphism; intrauterine growth retardation
**Leukodystrophy, adult onset, autosomal dominant**	LMNB1	Lamin B1	Slowly progressive; demyelination; autonomic dysfunction; pyramidal and cerebellar abnormalities
**Microcephaly 26, primary, autosomal dominant**			Microcephaly; developmental delay; variable intellectual development impairment
**Acquired partial lipodystrophy**	LMNB2	Lamin B2	Genetic susceptibility; bilateral and symmetric loss of subcutaneous fat from face, neck, upper extremities, thorax and abdomen
**Microcephaly 27, primary, autosomal dominant**			Microcephaly; Moderate to severe development delay; impaired intellectual development
**Progressive myoclonic epilepsy-9**			Seizures; progressive neurological decline; early ataxia
**Emery-Dreifuss muscular dystrophy 1, X-linked**	EMD	Emerin	Slowly progressive muscle weakness and atrophy; joint contractures; cardiomyopathy with conduction defects
**Emery-Dreifuss muscular dystrophy 4, autosomal dominant**	SYNE1	Nesprin-1	Variable phenotype with muscle weakness and atrophy; limb contractures
**Arthrogryposis multiplex congenita 3, myogenic type**			Hypotonia and muscle weakness; joint contractures; variable skeletal defects
**Spinocerebellar ataxia, autosomal recessive 8**			Progressive neurodegenerative disorder with variable onset and phenotype; gait and cerebellar ataxia
**Emery-Dreifuss muscular dystrophy 5, autosomal dominant**	SYNE2	Nesprin-2	Muscle weakness and atrophy; cardiac myopathy and arrhythmia
**Deafness, autosomal recessive 76**	SYNE4	Nesprin-4	Hearing loss
**Mandibuloacral dysplasia with type B lipodystrophy**	ZMPSTE24	Zinc Metalloproteinase STE 24	Craniofacial and skeletal abnormalities; generalized loss of subcutaneous adipose tissue; mottled pigmentation and atrophy of skin; short stature
**Restrictive dermopathy, lethal**			Early neonatal death; tight, rigid skin with erosion and fissures; joint contractures; superficial vessels; facial dysmorphism; intrauterine growth retardation
**Atypical Hutchinson-Gilford progeria**			Severe progeria
**Reynolds syndrome**	LBR	Lamin B receptor	Primary biliary cirrhosis; variable features of scleroderma
**Greenberg skeletal dysplasia**			Skeletal abnormalities; fetal death
**Pelger-Huet anomaly (PHA)**			Abnormal neutrophil nuclear shape and chromatin organization; homozygous mutation can cause PHA with mild skeletal abnormalities or Greenberg skeletal dysplasia

Muscles with mutations in the *LMNA* gene or absence of *LMNA* gene also display aberrant nuclear morphology, stability, mechanics, and DNA damage (Earle et al., 2020). Similarly, muscles with mutations in genes for LINC complex proteins such as emerin and nesprin-1 also display aberrant nuclear morphology, mechanics, and DNA damage (Fidzianska et al., 1998; Zhang et al., 2010). Mutations in the LINC complex proteins, such as SUN and nesprin, are disease modifiers that worsen the phenotype of laminopathies by further disrupting mechanotransduction pathways due to insufficient linkage to the cytoskeleton (Zhang et al., 2007; Meinke et al., 2014). Nuclear positioning is also impacted in muscles with mutations in lamin and nuclear envelope genes, with the appearance of central and clustered nuclei, as well as a reduced number of synaptic nuclei. The influence of the lamina and LINC appears to be bidirectional. That is, alterations in the lamina result in aberrant expression and organization of the LINC complex and the cytoskeleton. Conversely, mutations/deficiencies in the LINC complex or cell cytoskeleton can result in altered expression and organization of the nuclear lamina (Meinke et al., 2014). Thus, mutations of genes related to the nucleocytoskeleton pathway can severely impact mechanotransduction, and ultimately muscle function.

Proteins such as YAP and MLK1 are indicators reflecting nuclear mechanotransduction, with increased signaling in the nucleus in response to mechanical load. Interestingly, when lamin A is depleted, nuclear levels of both YAP and MLK1 are increased (Wiggan et al., 2020). Nuclear YAP is also increased in muscle stem cells with mutations in the *LMNA* and *SYNE-1* (gene for nesprin-1 protein) genes (Owens et al., 2020a). Conversely, overexpression of *LMNA* results in a decrease in nuclear YAP accumulation (Swift et al., 2013). While cyclic strain typically increases the nuclear accumulation of YAP (due to increased mechanical load), with mutations in *LMNA* gene, cyclic strain decreases the nuclear accumulation of YAP (Bertrand et al., 2014). Such studies support the notion of a link between lamin IFs and nucleo-cytoskeletal signaling. Recently, we and others observed increased YAP nuclear accumulation and signaling in aging skeletal muscles (Yoshida et al., 2019; Iyer et al., 2021), which also had a reduction in lamin-B1, but whether such findings are linked is still unknown.

Similar to YAP, ERK 1/2 signaling typically increases in skeletal muscle with mechanical loading, such as after exercise or repeated contractions (Kramer and Goodyear, 2007). Compared to healthy controls, ERK 1/2 signaling is increased in skeletal muscle with *LMNA* mutations (Muchir et al., 2007), and cortical neurons depleted of lamin B1 (Giacomini et al., 2016). Such results point to the nuclear lamina playing a pivotal role in nuclear mechanotransduction. Interestingly, inhibition of ERK 1/2 in skeletal muscle with *LMNA* mutations improves function (Muchir et al., 2013). Future studies will hopefully determine whether pharmacological interventions to nuclear mechanotransduction pathways can help ameliorate skeletal muscle weakness in laminopathies and sarcopenia.

## Nuclear Envelope and Exercise

Skeletal muscle has the ability to adapt to loading exercise, with consequent increases in muscle mass (hypertrophy) and strength. However, in muscles with mutations in the *LMNA* gene there is a reduction in the amount of hypertrophy, presumably due to abnormal mechanical signaling (Owens et al., 2020b). Mutations in the *LMNA* gene also result in cardiac dysfunction following exercise (Cattin et al., 2016). Patients with mutations in *LMNA* gene who are active have more cardiac dysfunction compared to patients with mutations in the *LMNA* gene who are sedentary. This could be due to mechanically weakened nuclei, which respond poorly to load, further exacerbating the dysfunction (Earle et al., 2020). Thus, further studies are needed to determine optimal exercise regimens for patients to maintain health and to avoid adverse outcomes.

Increasing stiffness of the extracellular matrix results in decreased phosphorylation of lamin A and consequently decreased degradation of lamin A (Cho et al., 2019). Skeletal muscle stiffness increases following loading exercise and injury (Green et al., 2012; Silver et al., 2021), however, the direct linkage between changes in lamin and exercise/injury in skeletal muscle remains unclear. Expression of nesprin-1, but not SUN-1, increases following endurance exercise in skeletal muscle (Bostani et al., 2020). This increase in nesprin-1 expression could be the result of myogenesis, which often occurs with endurance exercise (Abreu and Kowaltowski, 2020). However, in mature muscle fibers, nesprin-1 can be replaced in part by nesprin-2, while the SUN proteins remained unchanged during fiber maturation (Randles et al., 2010). Research into the role of the nuclear envelope in skeletal muscle exercise is still in its infancy and much more work is needed to further elucidate the relationship between nuclear mechanics and muscle performance.

## Conclusion

How mutations in genes for lamin IFs and NE proteins affect skeletal muscle function is complicated and studies are still being performed to understand the underlying mechanisms. Defects in the nuclear lamina, the LINC complex, and the NPCs can impair the ability of nuclei, and therefore the muscle cell, to respond appropriately to mechanical forces. Understanding the mechanisms of impaired mechanotransduction in aging and diseased muscle is likely to shed light on strategies designed to prevent and treat skeletal muscle dysfunction with disease and aging.

## Author Contributions

All authors listed have made a substantial, direct and intellectual contribution to the work, and approved it for publication.

## Conflict of Interest

The authors declare that the research was conducted in the absence of any commercial or financial relationships that could be construed as a potential conflict of interest.

## Publisher’s Note

All claims expressed in this article are solely those of the authors and do not necessarily represent those of their affiliated organizations, or those of the publisher, the editors and the reviewers. Any product that may be evaluated in this article, or claim that may be made by its manufacturer, is not guaranteed or endorsed by the publisher.
